# Molecular surveillance and phylogenetic traits of *Babesia bigemina* and *Babesia bovis* in cattle (*Bos taurus*) and water buffaloes (*Bubalus bubalis*) from Colombia

**DOI:** 10.1186/s13071-018-3091-2

**Published:** 2018-09-12

**Authors:** Jeiczon Jaimes-Dueñez, Omar Triana-Chávez, Andrés Holguín-Rocha, Alberto Tobon-Castaño, Ana M. Mejía-Jaramillo

**Affiliations:** 1 Grupo BCEI, Universidad de Antioquia UdeA, Calle 70 No. 52-21, Medellín, Colombia; 2 Grupo Malaria, Universidad de Antioquia UdeA, Calle 70 No. 52-21, Medellín, Colombia

**Keywords:** *Babesia*, Colombia, nPCR, Cattle, Water buffaloes

## Abstract

**Background:**

*Babesia bigemina* and *B. bovis* are two economically important hemoparasites affecting both cattle and buffaloes involved in dairy and beef production. In Colombia, although some parasitological and serological studies suggest an endemicity of these pathogens in areas under 1000 m, little is known about its molecular prevalence in different host. The objective of this study was to estimate the prevalence and molecular traits of these parasites in cattle and buffaloes from two Colombian regions.

**Methods:**

Between 2014 and 2016, a three-point longitudinal survey was designed in farms from Caribbean and Orinoquia regions to evaluate the molecular prevalence of *B. bigemina* and *B. bovis* using a nested PCR (n-PCR) targeting *hypothetical protein* (*hyp*) and *rhoptry-associated protein* (*RAP-1*) genes, respectively. A total of 1432 cattle, 152 buffalo and 1439 *Rhipicephalus microplus* samples were analyzed. Moreover, phylogenetic relationship of isolates was analyzed using the *18S* rRNA gene.

**Results:**

A molecular prevalence of 31.6% (24.2% for *B. bigemina* and 14.4% for *B. bovis*), 23.6% (6.5% for *B. bigemina* and 17.7% for *B. bovis*) and 4.3% (3.5% for *B. bigemina* and 1.0% for *B. bovis*) was observed in cattle, buffaloes and *Rhipicephalus microplus*, respectively. Higher values of infection were observed during the wet season and late wet season; nevertheless, other variables such as age, production type, sex, breed and babesiosis control were also significantly associated with infection. Prevalence analysis showed that *B. bovis* infection was higher in cattle that coexist with buffaloes, when compared to those which did not. For each species, phylogenetic analyses revealed a high genetic diversity of isolates without clusters related to the isolation source.

**Conclusions:**

To our knowledge, this is the first longitudinal survey that evaluates through molecular methods, the infection of *B. bigemina* and *B. bovis* in two important livestock regions from Colombia. This study reveals that the prevalence of infection by *Babesia* spp., in cattle and buffaloes are modulated by seasonal variations, host factors and vector traits. Our results provide new insights on the epidemiological aspects of infection of *Babesia* spp., in cattle and buffaloes, which must be taken into consideration when babesiosis control programs are implemented in the study area.

**Electronic supplementary material:**

The online version of this article (10.1186/s13071-018-3091-2) contains supplementary material, which is available to authorized users.

## Background

Babesiosis, caused by species of the genus *Babesia*, intraerythrocytic apicomplexan parasites, is a worldwide tick-borne hemoprotozoosis affecting many domestic and wild mammalian species. Two species are present in South America, *Babesia bovis* and *B. bigemina*, which are epidemiologically important in livestock and are biologically transmitted by the cattle tick *Rhipicephalus microplus* [1, 2]. Cattle with babesiosis usually show severe clinical signs that include fever, anemia, jaundice, loss of appetite, muscle tremors, haemoglobinuria and death [3], while in buffaloes clinical cases are rare and hardly associated with clinical signs such as fever, colic, anorexia and haemoglobinuria [4]. Although *B. bigemina* is more widespread, causing mortality rates up to 30% in animals without treatment, *B. bovis* is the most virulent *Babesia* parasite, generating mortality rates between 70–80%, as a result of the related neurological signs [5].

In Colombia, tick-borne diseases produce economic losses of approximately 4.2 million USD per year, and they affect mainly the cattle farms on the Atlantic coast, the Andean valleys, and the Orinoquia region, representing more than half of the national livestock population [6, 7]. Previous parasitological surveys of *Babesia* spp. carried out in these regions showed a prevalence of 1.1–3.8% in cattle [8–10], and 4.6–30.1% in buffaloes [11, 12], suggesting an important role of the latter host in maintaining the circulation of these pathogens. In Colombia the buffalo population is increasing with an annual growth up to 35%, most of them bred in the same pastures alongside the cattle [13]. In this sense, studies that evaluate the epidemiology and molecular features of *Babesia* spp. in both hosts have not been yet performed, ignoring whether the differences in the infection rate may be due to epidemiological conditions from study areas or to the particular traits of isolates in both hosts.

Although in South America several studies have shown seasonal variations of *R. microplus* [14, 15], in Colombia little is known about these variations in the infection rates of *Babesia* spp. in the livestock. Therefore, the aim of this study was to evaluate the transmission dynamic and genetic features of the causal agents of babesiosis in cattle and buffaloes from the Atlantic coast and Orinoquia regions, in order to understand the epidemiology of this disease in Colombian livestock and to contribute to more efficient control strategies.

## Methods

### Study area

**Fig. 1:**
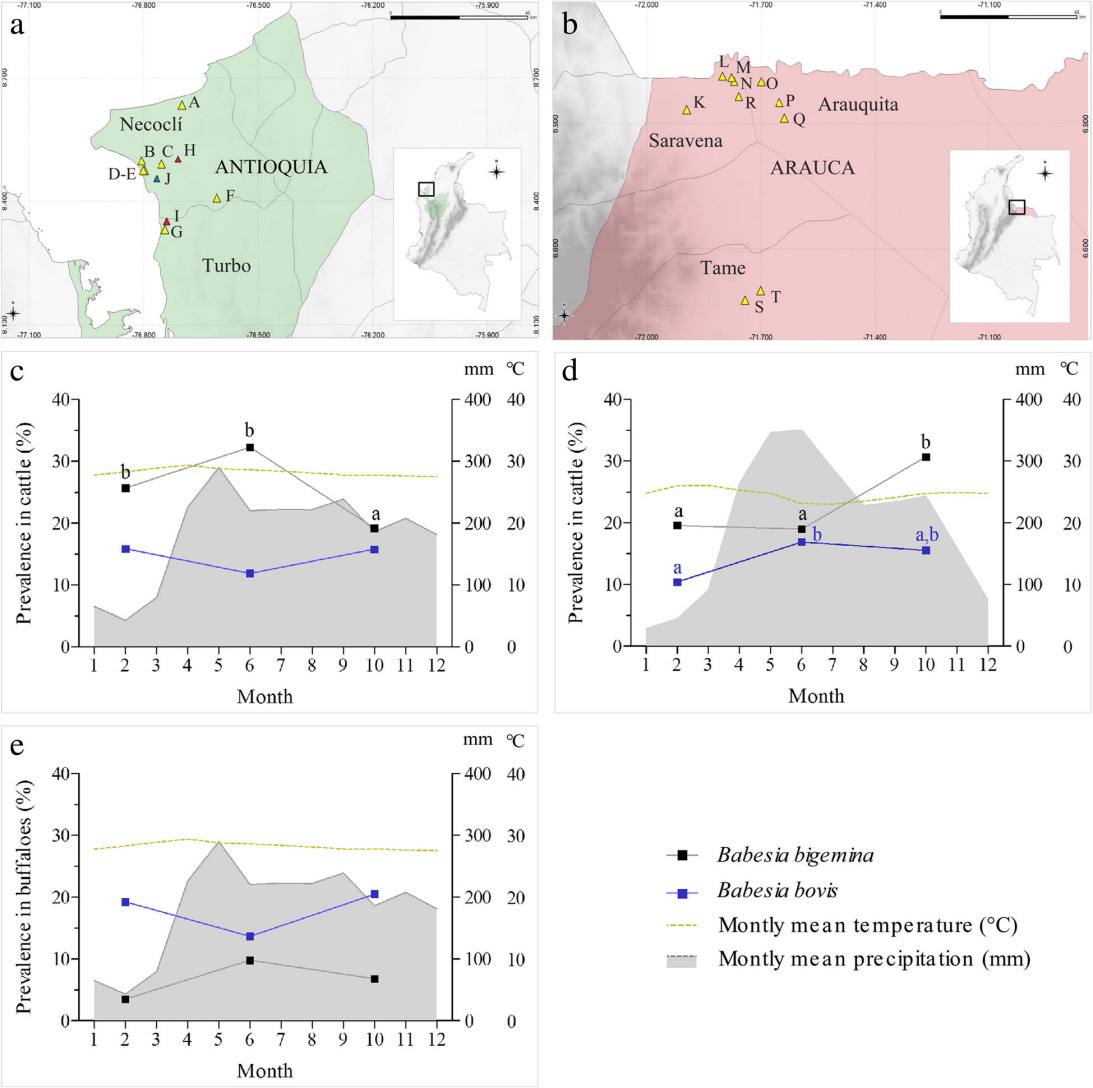
Map of Colombia showing the farms in Antioquia (**a**) and Arauca (**b**) where sampling was done, and the spatio-temporal prevalence of *B. bigemina* and *B. bovis* observed in cattle in Antioquia (**c**) and Arauca (**d**), and buffaloes in Antioquia (**e**). Yellow, blue and red triangles represent the farms where samples of cattle, buffaloes and both species were collected, respectively (see Additional file 1: Table S1 for additional information, according to uppercase farm code letter). Lowercase letters in the graphs represent significant differences assessed by the Chi-square test (*P* < 0.05)

The study was conducted from October 2014 to March 2016 on ten farms of the Antioquia department (A-J) (Fig. 1a) and ten farms of Arauca (K-T) (Fig. 1b), which represented 11.7 and 4.6% of the national livestock population, respectively [6]. The inclusion criteria for the farms included animal age records and restriction for the entry of livestock from other farms. The farms of Antioquia were located in the municipalities of Necoclí and Turbo in the Caribbean region, at altitudes under 8 m, with an annual biotemperature average of 26 °C and rainfall of 2400 mm, distributed over a dry season from January to March, a wet season from April to September, and a late wet season from October to December [16]. The farms of Arauca were located in the municipalities of Arauquita, Saravena and Tame in the Orinoquía region in the east of the country, at altitudes under 231 m, with an annual biotemperature average of 27 °C and rainfall of 2500 mm, distributed over a dry season from November to March, a wet season from April to July, and a late wet season from August to October [16].

Ninety-five percent of the farms employ extensive farming systems, where animals are fed with *Brachiaria* spp. and native grasses, and are at densities of 1.314 (SD = 0.359) livestock units (LUs) per hectare (ha), and the rest use the intensive farming system. The main species of cattle is *Bos indicus*, with breeds such as the Brahman, Gyr and Guzerat, and its crosses with *B. taurus*, including breeds such as Limousin, Brown Swiss and Holstein Friesians. For buffaloes, the main species is *Bubalus bubalis*, with breeds such as Carabao, Mediterranean, Murrah and their crosses. Babesiosis control is mainly based on vector control carried out with insecticides and acaricides such as avermectins, organophosphates and pyrethroids, as well as the use of antiprotozoal drugs such as imidocarb dipropionate and diminazine accurate. Additional information about the geographical location and characteristics of each farm is shown in Additional file 1: Table S1.

### Study design

According to previous epidemiological studies concerning seasonal variations of *R. microplus* and its relationship with transmission patterns of babesiosis [14, 17], a three-point longitudinal survey was designed to examine seasonal variations of *Babesia* spp*.* infection in the study area in cattle, buffaloes and ticks during the dry season, wet season and the late wet season.

### Sample size

The sample size was calculated using Epi Info™ 7.0, taking into account the number of cattle or buffalo in the farms of each department, a 22.5% probability of being infected according to Herrera et al. [9], a 95% confidence interval and a statistical error of 5%. Thus, the estimated sample size for cattle in each season was 220 and 260, in Arauca and Antioquia, respectively, while for buffaloes was 148 for Antioquia. Within each farm, the samples were collected randomly, considering the percentage of animals ≤ 1 year-old and >1 year-old according to Amorim et al. [18]. Cattle samples were collected on nine farms of Antioquia (A-I) (Fig. 1a) and ten farms of Arauca (K-T) (Fig. 1b), whereas buffalo samples were collected on three farms in Antioquia (H-J) (Fig. 1a).

### Blood sample collection and proccessing

For each animal, 5 ml of blood was collected from the coccygeal or jugular vein using EDTA.K3 Vacutainer tubes (Improve Medical, Guangzhou, China) and stored at 4 °C until processing. Additional information about the number of samples collected in each farm during each sampling is detailed in Additional file 1: Table S1. Blood samples were transferred within 6 h after collection into a glass micro-hematocrit capillary tube containing Na-heparin (80 IU/ml) (Vitrex Medical, Copenhagen, Denmark), sealed at one end with Cristaseal (Hawksley, Lancing, UK) and centrifuged for 5 min at 9000× *rpm* at room temperature, to measure the packed cell volume (PCV) in a micro-hematocrit capillary tube reader (Bacto, Sydney, Australia). Genomic DNA was extracted from 200 μl of blood using the Genomic DNA Purification Kit (Invisorb, Birkenfeld, Germany) according to the manufacturer’s instructions. Total DNA was diluted with 100 μl of elution buffer and stored at -20 °C until molecular diagnosis.

### Tick sample collection and processing

Between 5 and 20 ticks were collected from the highly infested animals selected for blood sampling along with others located on the same farms, using entomological forceps through examination of the perineum surface according to Baker & Ducasse [19]. All collected specimens were directly preserved in 70% ethanol and stored at 4 °C until processing. Tick samples were initially examined under a stereomicroscope (Leica Microsystems, Mannheim, Germany) and classified up to the species, sex and life stage according to morphological keys reported for ticks of veterinary importance in South America [20]. After that, specimens were grouped in pools of one to six individuals based on their life stage, sex, species and mammalian host. Considering that a positive correlation between the blood parasite levels of infected animals and the numbers of *Babesia* kinetes in the hemolymph of the dropped tick has been shown [21, 22], hemolymph DNA from each pool of ticks was extracted as detailed below. To that end, ticks were washed three times in 70% ethanol for 5 min to sterilize them [23], and subsequently, the legs were dissected in PBS as reported elsewhere [24]. This material was frozen and disrupted mechanically in liquid nitrogen [25] and then subjected to genomic DNA extraction using a DNeasy Blood & Tissue Kit (Qiagen, Hilden, Germany) according to the manufacturer’s instructions. Total DNA was diluted with 100 μl of elution buffer and stored at -20 °C until molecular diagnosis.

### Screening for the presence of *B. bigemina* and *B. bovis*

All DNA preparations were screened to test for the presence of *B. bigemina* and *B. bovis* using a nested PCR (n-PCR) targeting the *hypothetical protein* (*hyp*) and *rhoptry associated protein* (*RAP-1*) genes, respectively [26], which have been reported with a specificity of 100% and an analytical sensitivity corresponding to parasitemias of approximately 3.0 × 10^-2^ and 1.7 × 10^-2^ parasites/μl of the infected blood, respectively [27]. The oligonucleotide sequences used in the present study and their annealing temperatures are shown in Table 1. All PCR assays were conducted in 25 μl reactions containing 1× reaction buffer (100 mM Tris-HCl, 50 mM KCl, pH 8.8), 0.2 mM dNTP, 1.5 mM MgCl_2_, 0.4 μM of each primer, 0.625 UI of *Taq* DNA polymerase (Thermo Scientific, Massachusetts, USA), and 5 μl (100 ng) of DNA samples for the first PCR and 1 μl of PCR product for the second. PCR products were analyzed by 2% agarose gel electrophoresis, stained with GelRed (Thermo Fisher Scientific) and visualized under UV light. Sample preparations were considered positive for *B. bigemina* or *B. bovis* when a band of 170 or 290 bp, respectively, was observed in the electrophoresis of the second PCR. To confirm the specificity of the molecular diagnosis, the PCR product of one positive sample of *B. bigemina* and one of *B. bovis* were purified and sequenced using the Sanger method at Macrogen, Korea.

**Table 1:** List of primers used in PCR assays

Organism	Target gene	Primer	Oligonucleotide sequence (5′-3′)	Amplicon size (bp)	Annealing T (°C)	Reference
*B. bigemina*	*hyp*	BiIA	CATCTAATTTCTCTCCATACCCCTCC	278	63	[26]
		BiIB	CCTCGGCTTCAACTCTGATGCCAAAG			
		BiIAN	CGCAAGCCCAGCACGCCCCGGTGC	170	70	
		BiIBN	CCGACCTGGATAGGCTGTGTGATG			
*B. bovis*	*RAP-1*	BoF	CACGAGGAAGGAACTACCGATGTTGA	350	61	[26]
		BoR	CCAAGGAGCTTCAACGTACGAGGTCA			
		BoFN	TCAACAAGGTACTCTATATGGCTACC	290	67	
		BoRN	CTACCGAGCAGAACCTTCTTCACCAT			
*Babesia* spp.	*18S rRNA*	A	ACCTGGTTGATCCTGCCAG	~1700	60	[30]
		B	GATCCTTCTGCAGGTTCACCTAC			
*B. bigemina*	*18S rRNA*	Bbig200F	GCGTTTATTAGTTCGTTAACC	~1200	56	[32]
		Bbig1400R	ACAGGACAAACTCGATGGATGC			
*B. bovis*	*18S rRNA*	AN	GCTTGTCTTAAAGATTAAGCCATGC	~1550	60	[31]
		BN	CGACTTCTCCTTCCTTTAAGTGATAAG			
Mammalian	*cyt b*	Cyt bF	CCCCTCAGAATGATATTTGTCCTCA	~383	60	[28]
		Cyt bR	CCATCCAACATCTCAGCATGATGAAA			
Insects	*cox*1	LCOI490	GGTCAACAAATCATAAAGATATTGG	~710	45	[29]
		HCO2198	TAAACTTCAGGGTGACCAAAAAATCA			

*Abbreviation*: *T* temperature

Finally, to check the presence of PCR inhibitors and amplifiable DNA, a 383 bp fragment from the *cyt b* gene from mammals [28], and a 710 bp fragment from the *cox*1 gene of arthropods [29], were amplified by PCR in all negative blood and tick samples, respectively (Table 1). Samples that did not amplify were excluded from the data analysis.

### Amplification and sequencing of genetic markers

After molecular diagnosis, the strongest positive samples for the amplification of *hyp* and *RAP-1* genes were selected to amplify the *18S* rRNA gene from *B. bigemina* and *B. bovis*. The complete *18S* rRNA gene of both species was initially amplified by PCR using oligonucleotides A and B [30], and 1 μl of PCR product was used as template in the n-PCR using oligonucleotides set AN/BN [31], and Bbi200F/Bbi1400R [32] for each species, respectively. The amplified fragments were separated in 2% agarose gels stained with GelRed (Thermo Fisher Scientific), excised from the gels, purified by a Zymoclean™ Gel DNA Recovery Kit (Zymo Research, Irvine, CA, USA) and cloned using the pGEM-T Easy vector system (Promega, Wisconsin, USA) according to the manufacturer’s instructions. One to two clones from each isolate were purified and completely sequenced in both strands using the Sanger method at Macrogen.

### Phylogenetic analyses

Sequences were aligned using CLUSTALW [33] as implemented in BioEdit v.7.1.9 [34]. For each species, phylogenetic analyses were performed using sequences obtained in this study along with reference sequences of *Babesia* spp. derived from cattle, buffaloes and vaccination strains from America and other continents. Each alignment was analyzed using the Maximum Composite Likelihood method with Kimura 2-parameters model in MEGA6.0 software. Internal branch confidence was assessed by the boot-strapping method using 1000 bootstrap replicates. The trees were drawn with FigTree v.1.4.2 (Institute of Evolutionary Biology, University of Edinburgh, UK).

### Data source

The environmental dataset used in this study corresponded to monthly values of precipitation (mm) and mean temperature (°C) from 2011 to 2016 in the study areas, which were provided by the Institute of Hydrology, Meteorology and Environmental Studies of Colombia (IDEAM). The meteorological stations (12045010, 12030020 and 12025030) from Antioquia and those of Arauca (36030030, 37045010 and 36025010) were chosen according to their proximity to the study farms.

### Data analysis

To construct models that could explain the dependent variable (*B. bigemina* or *B. bovis* infection) as a function of 11 qualitative independent variables related to host and environmental conditions (Additional file 1: Table S2), generalized estimated equation (GEE) models were run using a binomial distribution, a *log link* function and exchangeable interclass correlation. In all analyses the feature farm was included as the group variable. Initially, the independent variables were subjected to univariate analysis. Those with a statistical association (here considered to be *P* < 0.25, Chi-square test, according to Hosmer & Lemeshow [35]) were tested in the multivariate model using a stepwise method. The level of statistical significance of the variables included in the multivariate model was set at *P* < 0.05. The results are expressed as Exp (B) (an estimate of the prevalence ratio, PR) and 95% CI. Additional prevalence comparisons were performed using the Chi-square test.

The cut-off to determine anemic cattle and buffaloes was PCV ≤ 24% and ≤ 29%, respectively, according to described by Abd Ellah et al. [36] and Rockett & Bosted [37]. Finally, the relationship between *Babesia* spp. prevalence in both mammalian hosts with the age was evaluated through correlation analysis, after a Shapiro-Wilk normality test. *P* < 0.05 was considered significant. The sizes of age groups varied from 4 to 12 % of the total population. Data analyses were performed using SPSS v.18.0 statistical software.

## Results

### *Babesia* spp. infection is modulated by host and environmental conditions

A total of 1432 cattle (715 from Arauca and 717 from Antioquia) and 152 buffaloes were analyzed, which corresponded to 75.0 and 79.6% of animals > 1 year-old, with a mean age of 4.69 (SD = 3.64) and 6.42 years (SD = 5.96), respectively. Of the total cattle analyzed, 31.6% (453/1432) were positive for *Babesia* spp. of which 24.2% (347/1432) and 14.4% (207/1432) were positive for *B. bigemina* and *B. bovis*, respectively. For buffaloes, 23.6% (36/152) were positive for *Babesia* spp. of which 6.5% (10/152) and 17.7% (27/152) were positive for each species, respectively. The prevalence of co-infections (*B. bigemina* + *B. bovis*) for cattle and buffaloes was 7.0% (101/1432) and 0.6% (1/152) (Table 2), respectively.

**Table 2:** Prevalence of *Babesia bigemina* and *Babesia bovis* determined by molecular techniques in cattle, buffaloes and ticks from Colombia, during dry, wet and late wet seasons

Detected pathogens	Dry season		Wet season		Late wet season		Annual	
	*n*	Positive (%)	*n*	Positive (%)	*n*	Positive (%)	*n*	Positive (%)
*B. bigemina*								
Cattle	466	105 (22.5)	463	118 (25.4)	503	124 (24.6)	1432	347 (24.2)
Buffaloes	57	2 (3.5)	51	5 (9.8)	44	3 (6.8)	152	10 (6.5)
*R. microplus* (pools)	45	2 (4.4)	23	0 (0.0)	210	8 (3.8)	278	10 (3.5)
*D. nitens* (pools)	7	–	–	–	4	0 (0)	11	0 (0)
*A. cajennense* (*s.l.*) (pools)	–	–	–	–	9	0 (0)	9	0 (0)
*B. bovis*								
Cattle	466	61 (13.0)	463	67 (14.4)	503	79 (15.7)	1432	207 (14.4)
Buffaloes	57	11 (19.3)	51	7 (13.7)	44	9 (20.5)	152	27 (17.7)
*R. microplus* (pools)	45	0 (0)	23	0 (0)	210	3 (1.4)	278	3 (1.0)
*D. nitens* (pools)	7	–	–	–	4	0 (0)	11	0 (0)
*A. cajennense* (*s.l.*) (pools)	–	–	–	–	9	0 (0)	9	0 (0)
*B. bigemina + B. bovis*								
Cattle	466	25 (5.3)	463	35 (7.5)	503	41 (8.1)	1432	101 (7.0)
Buffaloes	57	0 (0)	51	0 (0)	44	1 (2.2)	152	1 (0.6)
*R. microplus* (pools)	45	0 (0)	23	0 (0)	210	1 (0.4)	278	1 (0.3)

**Fig. 2:**
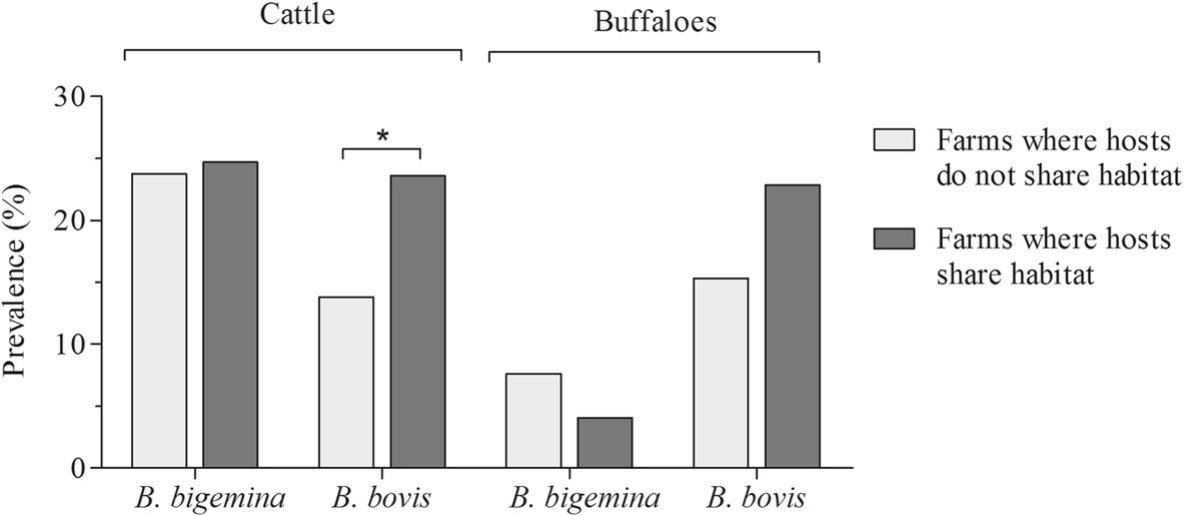
Comparison of the prevalence of *B. bigemina* and *B. bovis* in animals who did not share habitat (cattle or buffaloes) compared with animals sharing habitat (cattle and buffaloes). *Significant differences assessed by the Chi-square test (*P* < 0.05)

Interestingly, during the three samplings, *B. bigemina* was the most prevalent species for cattle (Fig. 1c, d), while for buffaloes *B. bovis* was most prevalent (Fig. 1e). The highest *Babesia* spp. prevalence values for both cattle and buffaloes were observed during the wet season or late wet season, with significant seasonal variations found in cattle from both departments (Fig. 1c, d). *B. bovis* infection was higher in cattle that coexist with buffaloes, when compared to those who did not (*P <* 0.05) (Fig. 2).

**Fig. 3:**
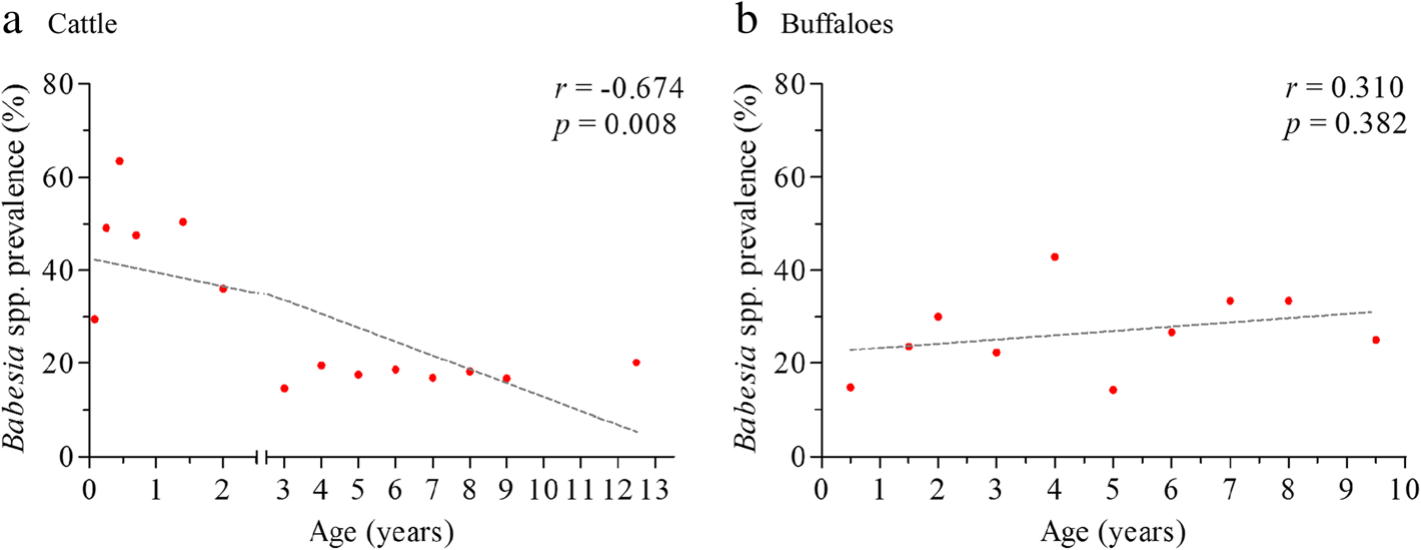
Correlation analysis between the prevalence of *Babesia* spp. and age groups of cattle (**a**) and buffaloes (**b**) from the study area. A significant negative Pearson’s correlation coefficient (*r* = -0.674) was observed for cattle

In cattle, PCV analyses showed a mean of 33.7% (SD = 5.7%), 29.6% (SD = 6.4%) and 31.1% (SD = 6.01%) for *Bos indicus*, *B. taurus* and their crosses, respectively, while for buffaloes the mean PCV was 33.5% (SD = 7.13%). A proportion of 8.9% (123/1379) and 33.5% (51/152) of cattle and buffaloes showing anemia signs was observed, respectively. Both *B. bigemina* and *B. bovis* prevalence was higher in animals with normal PCV values than those with anemia signs (Additional file 1: Table S2). Significant negative Pearson’s correlation coefficients (Spearman’s *r* = -0.674, *P* = 0.008) was observed between *Babesia* spp. prevalence and the age range in cattle, with lower prevalence of infection in older animals (Fig. 3a). On the contrary, no significant correlation was observed in the case of buffaloes (Fig. 3b). All cattle and buffalo negative samples amplified *cyt b* gene. Finally, the *hyp* gene 128 bp derived from Arauca’s cattle (Additional file 2: nucleotide sequence) showed 95% identity with scaffold 71,030 of the *B. bigemina* genome (LK055111.1), while the 267 bp of the *RAP-1* gene from Antioquia’s cattle (KX365053) showed 100% identity with the R1A strain of *B. bovis* identified in Argentina (AF030062.1).

### Molecular analysis detected a low infection rate of *Babesia* spp. in *R. microplus*

Of 1479 ticks collected from 216 bovines across the three sampling periods in both departments, 97.2% (1439/1479) corresponded to *R. microplus*, 1.8% (27/1479) to *Dermacentor nitens* and 0.9% (13/1479) to *Amblyoma cajennense* (*sensu lato*). Regarding life stages, 97.5% of *R. microplus* samples were adults (89.6% females and 10.4% males) and 2.5% nymphs; for *D. nitens* 88.9% were adults (100% females) and 11.1% nymphs, while for *A. cajennense* (*s.l*.) 60.0% were adults (100% females) and 40.0% nymphs. Of the total of pools of *R. microplus* analyzed, 4.3% (12/278) were positive for *Babesia* spp. of which 3.5% (10/278), 1.0% (3/278) and 0.3% (1/278) were positive for *B. bigemina*, *B. bovis*, and co-infected, respectively (Table 2). All positive pools corresponded to engorged female, without significant differences in the infection rate between both departments. *D. nitens* and *A. cajennense* (*s.l.*) were negative for both *B. bigemina* and *B. bovis*. All ticks negative samples had an amplified *cox*1 gene.

### *Babesia* spp. infection in cattle and buffaloes is influenced by different variables

**Fig. 4:**
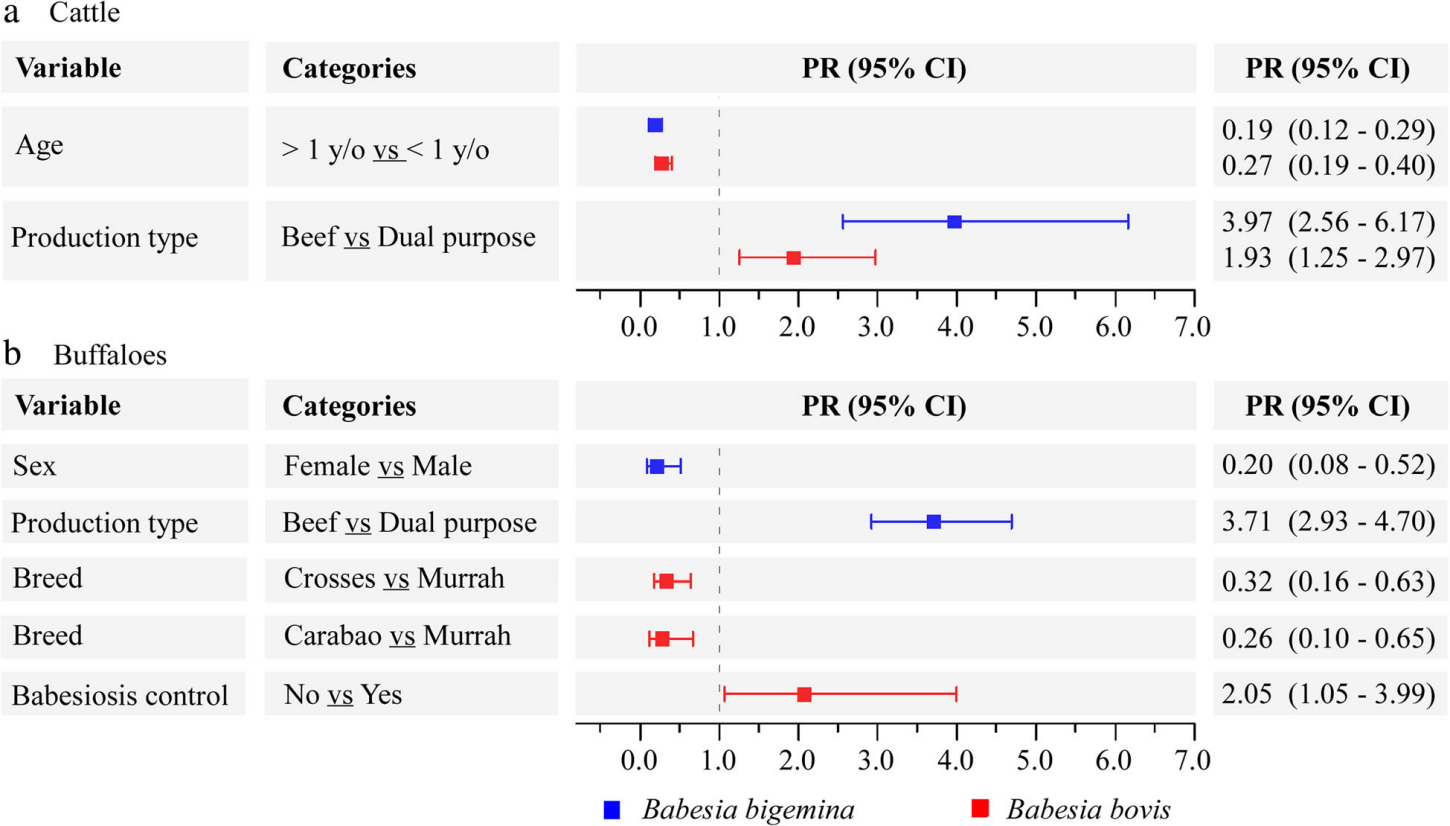
Forest plot of prevalence ratio (PR) obtained from the multivariate model (GEE) of infection with *B. bigemina* and *B. bovis* in cattle (**a**) and buffaloes (**b**) from Colombia. All prevalence ratios of independent variables are discriminated by category comparison. Squares and horizontal bars represent PRs and 95% CIs, respectively

In univariate analysis, the detection of at least one *Babesia* spp.-positive cattle by molecular techniques was statistically associated (*P* < 0.25) with all independent variables, except for breed, PCV, sampling season and department for *B. bigemina*; and breed, sampling season, department, farming system and vector control for *B. bovis* (Additional file 1: Table S2). However, in the multivariate analysis, only age and production type, were significantly associated (*P* < 0.05) with the infection of both *B. bovis* and *B. bigemina* (Fig. 4a).

For the case of buffaloes, univariate analysis showed statistical associations (*P* < 0.25) of *Babesia* spp. infection with all independent variables except for sex, PCV, sampling season, ambiental temperature, babesiosis control and municipality for *B. bigemina*; and PCV, sampling season, ambient temperature and production type for *B. bovis* (Additional file 1: Table S2). However, in the multivariate analysis, only sex and production type were significantly associated (*P* < 0.05) with the infection of *B. bigemina*, and breed and babesiosis control were significantly associated with the infection of *B. bovis* (Fig. 4b).

### Phylogenetic analyses of *Babesia* spp. do not show differentiation between isolation sources

**Fig. 5:**
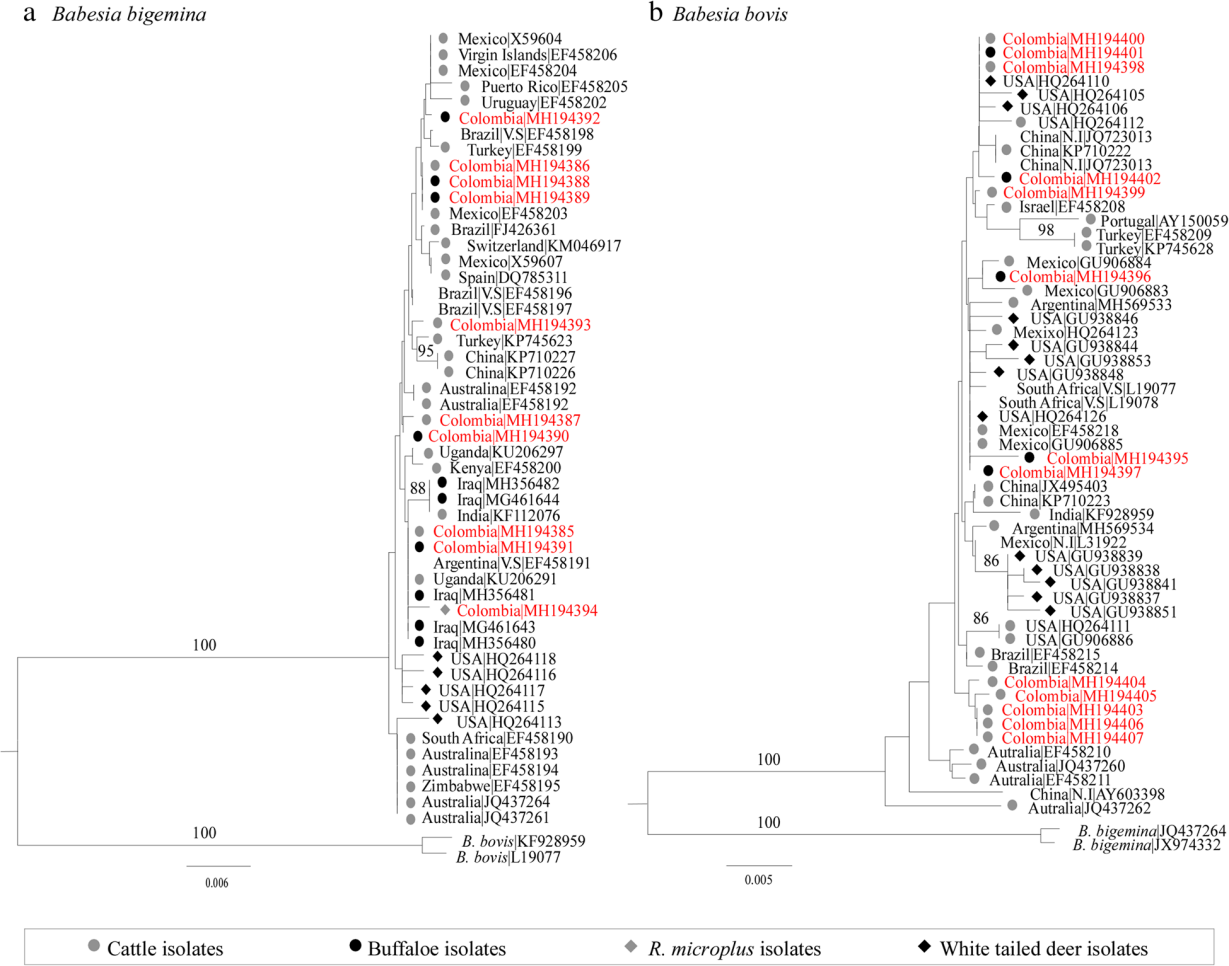
Maximum likelihood tree based on *18S* rRNA genes of *B. bigemina* (**a**) and *B. bovis* (**b**) isolates. Taxa in red represent the sequences of isolates reported in this paper. Node support bootstrap values > 80% are shown on the branches. *Abbreviations*: N.I, non-identified source; V.S, vaccination strain

A total of 10 (MH194385-MH194394) sequences of *B. bigemina* derived from PCR products cloned into the pGEM-T Easy vector system (Promega) from 4 cattle, 5 buffaloes and 1 pool of ticks (*R. microplus*), and 13 (MH194395-MH194407) sequences of *B. bovis* from 8 cattle and 5 buffaloes were analyzed. Both *B. bigemina* and *B. bovis* phylogenetic trees showed clusters with high proximity of the Colombian isolates to world isolates derived from different hosts. Moreover, clusters related to each isolation source were not observed in each species (Fig. 5a, b).

## Discussion

*Babesia bigemina* and *B. bovis* are the two economically important species affecting livestock farms involved in dairy and beef production in Colombia [1, 10, 12]. Given the paucity of studies on the distribution and characterization of these pathogens in different mammalian hosts, control strategies have been unsuccessful. To our knowledge, this is the first longitudinal survey and molecular characterization of *B. bigemina* and *B. bovis* in cattle and buffaloes from livestock areas in Colombia to consider eco-epidemiological variables associated with infection; this can improve the epidemiological understanding and control strategies of babesiosis in this country.

The results reported herein demonstrate that in the study area, the two *Babesia* species exhibited differences in prevalence between the mammalian hosts studied, with *B. bigemina* being most prevalent in cattle (24.2%) and *B. bovis* in buffaloes (17.7%). Similar results were observed in livestock areas from Argentina, Brazil and Colombia, in which the molecular prevalence of *B. bigemina* and *B. bovis* in cattle was 46.4 ± 7.0% and 11.7 ± 3.6%, respectively [10, 18, 38–40], while in buffaloes it was 8.6 ± 7.6% and 17.5 ± 8.8% for each species, respectively [41–43]. These findings, together with those obtained from the spatio-temporal analysis, in which these proportions did not vary with time (Fig. 1c-e), suggest that South American buffalo populations tend to support a higher prevalence of *B. bovis* compared to cattle populations.

The *18S* rRNA gene has been previously used to identify genetic clusters of *Babesia* spp. associated with specific hosts [32]. Considering the paucity of phylogenetic studies of *Babesia* spp. in livestock from South America, a phylogenetic approach to Colombian isolates was applied. Interestingly, our results showed that *Babesia* spp. isolates derived from cattle and buffaloes are not associated with particular clusters, suggesting a lack of genetic structure in these isolates and a possible circulation of strains between both hosts. These results, together with the higher prevalence of *B. bovis* observed in cattle coexisting with buffaloes (Fig. 2), support the idea that buffaloes might act as reservoir hosts for the infection of *B. bovis* in cattle. Therefore, special management measures have to be taken to prevent economic losses related to infection of this pathogen in cattle populations such as decreasing the tick transfer between cattle and buffaloes on farms with both hosts, as well as sharing of needles between them. On the other hand, the lack of genetic structure could be due to a variability of apicomplexan parasites that has been proposed as a survival strategy against the vertebrate immune response [44–46]. In this sense, genetic diversity observed here could be associated with continuous immune pressure by both hosts. Nevertheless, other factors, such as multiple introductions of strains from various geographical locations, and the diversity of biological and mechanical vectors, could also explain this behavior.

According to Smith et al. [47] enzootic stability of *Babesia* spp. is maintained when at least 75% of the herd is infected before nine months of age. On this matter, experimental and epidemiological studies in South America have provided evidence for significant differences between molecular and serological diagnosis of *Babesia* spp. showing that prevalence is two to three times more in indirect-ELISA compared to PCR analysis [41, 48]. Thus, the active infection rate detected by molecular methods in cattle under six months old (> 60%) together with the lack of anemic signs associated (Fig. 3a), suggest an enzootic stability in this population, as observed in other livestock areas from Colombia [10, 12, 49]. However, future studies using serological test are necessary to confirm the enzootic stability in the study area.

Although buffaloes did not show clinical signs associated with infection, the prevalence was variable between age groups (Fig. 3b), as previously observed in endemic areas of South America [11, 50]. In Colombia, like in other South American countries, buffalo populations have increased notably during recent years from approximately 100,000 buffaloes in 2010 to 250,000 in 2016 [6, 13]. This is mainly due to changing approaches towards meat and milk production, and to the perception that buffaloes are more tolerant than cattle to infection with *Babesia* spp. and the subsequent acute clinical effects resulting from such infections [51, 52]. This tendency, which included movement of animals to new areas with different transmission rates, could explain the heterogeneous dynamic of the prevalence in the age groups and the lack of clinical signs observed in these hosts; nevertheless, the low number of farms and blood samples evaluated for this species and the use of molecular techniques also could explain these results.

In Colombia, ecological studies that evaluated the dynamics of *R. microplus* at altitudes under 600 m showed that high infestation rates in cattle were observed in the days after the rain peaks, when the humidity and temperature conditions were optimal for the development of all tick life stages [53, 54]. Interestingly, our results showed that although *B. bigemina* and *B. bovis* were found during all samplings, seasonal variations were observed, with high frequencies detected during wet and late wet season in both hosts (Fig. 1c-e). This suggests that ticks and babesiosis control systems should be intensified during these seasons to reduce the economic impact resulting from outbreaks associated with the high transmission of these pathogens during the humid months.

In South America, little is known about the infection rates of *Babesia* spp., established by molecular methods, in *R. microplus*. Epidemiological studies that evaluated this feature in endemic areas from Brazil [27, 55] and Colombia [40], showed molecular infection rates ranging between 16.8–88.6%. Surprisingly, our results showed that infection rates in *R. microplus* were lower than 5.0% during all samplings (Table 2), indicating a discrepancy with those previously reported. Given that samples analyzed in previous studies correspond to DNA obtained from the whole body of the specimen, it is likely that infestation rates detected by them correspond to parasite stages or DNA present in the midgut, and not specifically to parasites in the hemolymph of the female tick. However, other factors such as levels of parasitemia in the hosts, which can modulate the infection rates in tick populations [21], as well as differences in the sensitivity of molecular diagnostic methods, cannot be ruled out. With regard to the high proportion of *B. bigemina* detected in the hemolymph compared to *B. bovis* (Table 2), this may be due to the ability of *B. bigemina* to remain through generations of *Rhipicephalus* spp. in the absence of reinfection, which does not happen in *B. bovis* [21, 56]; nevertheless, the high degree of pathogenicity of *B. bovis* in cattle, which lead to parasitemia less that 1% during acute infection [57], may also explain these results.

Although, the *Babesia* spp. infection is mainly associated with tick exposure [2, 55], its dynamics is complex and depends on many factors, whose association determines the prevalence of infection in the livestock. Herein, we identified different variables related to *Babesia* spp. infection, which could be modulating the prevalence of infection in the study area. In cattle, both *B. bigemina* and *B. bovis* infection were specifically associated with the age and production type (Fig. 4a). As discussed previously, the lower probability of adults of being infected with *Babesia* spp. when compared to calves is explained by the apparent enzootic stability in the study area, in which the higher infection rate observed in calves give rise to a fully protected adult herd with little chance of developing clinical signs against new infections [48, 58]. In the same way, the significantly higher infection rate of *Babesia* spp. observed in young animals can be explained by the fact that they have a softer skin compared to adults, which facilities the mouth-part penetration of the vector, making them the preferred host of ticks [59]. These findings, together with the low infection rate of *Babesia* spp. detected in ticks and the poor transovarial transmission of these pathogens in *R. microplus* (< 50%) [21, 60] suggest that a high tick infestation rate in young cattle is necesary to produce the high infection rate detected in this age group. On the other hand, the protective effect observed both in cattle and buffaloes dedicated to dual-purpose (Fig. 4a, b) suggest that frequent hygiene practices and tendency to use adult females in this group, could explain their low prevalence compared with those dedicated only to beef production. As a consequence, improving the implementation of vector and vector-borne pathogen control systems in animals dedicated to beef production will prevent sporadic outbreaks of babesiosis in these productions type. Finally, other variables such as sex, breed and babesiosis control were differentially associated with *Babesia* spp. infection in buffaloes (Fig. 4b). These findings showing a higher probability of infection in males, animals of the Murrah breed, and those kept on farms without babesiosis control systems, could encourage farmers from the study area to intensify these in buffalo populations.

## Conclusions

To our knowledge, this is the first epidemiological study investigating the spatio-temporal prevalence of *B. bovis* and *B. bigemina* in cattle, buffaloes and ticks, from two important livestock areas of Colombia. Our results revealed that the prevalence of infection by *Babesia* spp., in cattle and buffaloes are modulated by seasonal variations, host factors and vector traits. Furthermore, while in cattle the infection occurs under enzootic stability, in buffaloes it could be associated with an enzootic instability, accompanied by a high prevalence of *B. bovis* which suggests an important role of these animals as possible reservoir hosts of infection for cattle.

## Additional files

Additional file 1: **Table S1.** Geographical location and characteristics of the farms evaluated in Antioquia and Arauca, Colombia. **Table S2.** Bivariate model of infection with *Babesia* spp., in livestock herds from Antioquia and Arauca, Colombia. (DOCX 58 kb)

Additional file 2: Nucleotide sequences derived from a hypothetical protein of *Babesia bigemina.* (FAS 1 kb)

## Abbreviations

rRNA: Ribosomal ribonucleic acid
SD: Standard deviation
