# Supplementary material for: Molecular surveillance and phylogenetic traits of Babesia bigemina and Babesia bovis in cattle (Bos taurus) and water buffaloes (Bubalus bubalis) from Colombia
Source: Parasit Vectors. 2018 Sep 12;11:510. doi: 10.1186/s13071-018-3091-2 (PMC6136160; doi:10.1186/s13071-018-3091-2)
Supplement: Supplementary file 1 — Table S1. Geographical location and characteristics of the farms evaluated in Antioquia and Arauca, Colombia. Table S2. Bivariate model of infection with Babesia spp., in livestock herds from Antioquia and Arauca, Colombia. (DOCX 58 kb) [file 13071_2018_3091_MOESM1_ESM.docx]

**Additional file 1: Table S1. Geographical location and characteristics of the farms evaluated in Antioquia and Arauca, Colombia.**

| Farm | Latitude | Longitude | Department | Municipality | Population | Livestock units (LUs)/hectare | Samples collected | | | |
| --- | --- | --- | --- | --- | --- | --- | --- | --- | --- | --- |
|  |  |  |  |  |  |  | Dry season | Wet season | Late wet season | Total |
| Cattle samples description | | | | | | | | | | |
| A | 863.423 | -7.669.935 | Antioquia | Necoclí | 1900 | 1.73 | 30 | 30 | 31 | 91 |
| B | 849.876 | -7.680.486 | Antioquia | Necoclí | 800 | 1.43 | 31 | 43 | 30 | 104 |
| C | 849.028 | -7.681.940 | Antioquia | Necoclí | 450 | 0.92 | 32 | 24 | 31 | 87 |
| D | 847.538 | -7.680.084 | Antioquia | Necoclí | 200 | 1.64 | 29 | 30 | 30 | 89 |
| E | 847.472 | -7.679.691 | Antioquia | Necoclí | 1700 | 1.39 | 0 | 33 | 28 | 61 |
| F | 840.752 | -7.660.850 | Antioquia | Necoclí | 1500 | 1.75 | 30 | 36 | 33 | 99 |
| G | 833.180 | -7.674.387 | Antioquia | Turbo | 1800 | 1.84 | 30 | 30 | 33 | 93 |
| H | 850.295 | -7.670.964 | Antioquia | Necoclí | 300 | 1.13 | 29 | 0 | 30 | 59 |
| I | 835.184 | -7.673.902 | Antioquia | Turbo | 200 | 1.12 | 15 | 0 | 19 | 34 |
| Total | | | | | | | 226 | 226 | 265 | 717 |
| K | 693.365 | -7.189.498 | Arauca | Saravena | 200 | 1.23 | 25 | 26 | 0 | 51 |
| L | 701.457 | -7.180.067 | Arauca | Saravena | 110 | 1.12 | 30 | 29 | 28 | 87 |
| M | 701.122 | -7.177.768 | Arauca | Saravena | 50 | 1.36 | 15 | 18 | 22 | 55 |
| N | 700.203 | -7.177.016 | Arauca | Saravena | 130 | 1.18 | 31 | 24 | 27 | 82 |
| O | 700.185 | -7.169.867 | Arauca | Arauquita | 90 | 0.82 | 30 | 29 | 24 | 83 |
| P | 695.110 | -7.165.200 | Arauca | Arauquita | 160 | 1.14 | 27 | 27 | 33 | 87 |
| Q | 691.465 | -7.163.848 | Arauca | Arauquita | 130 | 1.64 | 28 | 24 | 27 | 79 |
| R | 696.511 | -7.175.786 | Arauca | Saravena | 140 | 0.95 | 29 | 35 | 30 | 94 |
| S | 647.614 | -7.174.180 | Arauca | Tame | 110 | 0.75 | 0 | 0 | 25 | 25 |
| T | 649.795 | -7-170.067 | Arauca | Tame | 100 | Confinement | 25 | 25 | 22 | 72 |
| Total | | | | | | | 240 | 237 | 238 | 715 |
| Buffaloes samples description | | | | | | | | | | |
| H | 850.295 | -7.670.964 | Antioquia | Necoclí | 60 | 1.34 | 10 | 11 | 9 | 30 |
| I | 835.184 | -7.673.902 | Antioquia | Turbo | 90 | 1.23 | 15 | 10 | 14 | 39 |
| J | 845.500 | -7.676.521 | Antioquia | Necoclí | 180 | 1.11 | 32 | 30 | 21 | 83 |
| Total | | | | | | | 57 | 51 | 44 | 152 |

**Additional file 1: Table S2. Bivariate model of infection with *Babesia* spp., in livestock herds from Antioquia and Arauca, Colombia.**

| Cattle | | | | Buffaloes | | | |
| --- | --- | --- | --- | --- | --- | --- | --- |
| Independent variables | Number of samples | Positive (%) | *χ2; df; P* | Independent variables | Number of samples | Positive (%) | *χ2; df; P* |
| *B. bigemina* |  |  |  |  |  |  |  |
| Age |  |  |  | Age |  |  |  |
| ≤ 1 year old | 358 | 160 (44.6) | **100.5;1;0.000** | ≤ 1 year old | 31 | 4 (12.9) | **2.5;1;0.111** |
| > 1 year old | 1074 | 187 (17.4) |  | > 1 year old | 121 | 6 (4.9) |  |
| Sex |  |  |  | Sex |  |  |  |
| Female | 1059 | 210 (19.8) | **40.5;1;0.000** | Female | 135 | 7 (5.1) | 3.8;1;0.051 |
| Male | 373 | 137 (36.7) |  | Male | 17 | 3 (17.6) |  |
| Breed |  |  |  | Breed |  |  |  |
| BI | 740 | 182 (24.5) | 0.5;2;0.749 | Carabao | 34 | 1 (2.9) | **7.1;3;0.067** |
| BI x BT | 297 | 67 (22.5) |  | Murra | 30 | 0 (0.0) |  |
| BT | 395 | 98 (24.8) |  | Mediterranean | 37 | 2 (5.4) |  |
|  |  |  |  | Crosses | 51 | 7 (13.7) |  |
| PCV |  |  |  | PCV |  |  |  |
| Anemic | 123 | 27 (21.9) | 20.9;1;0.830 | Anemic | 51 | 2 (3.9) | 0.8;1;0.348 |
| Normal | 1256 | 292 (23.2) |  | Normal | 101 | 8 (7.9) |  |
| Sampling season |  |  |  | Sampling season |  |  |  |
| Dry | 466 | 105 (22.5) | 1.1;2;0.555 | Dry | 57 | 2 (3.5) | 1.7;2;0.419 |
| Wet | 463 | 118 (25.4) |  | Wet | 51 | 5 (9.8) |  |
| Late wet | 503 | 124 (24.5) |  | Late wet | 44 | 3 (6.8) |  |
| Deparment |  |  |  | Deparment |  |  |  |
| Antioquia | 717 | 182 (25.3) | 1.0;1;0.308 | Antioquia | 152 | 10 (6.5) | - |
| Arauca | 715 | 165 (23.0) |  | - | - |  |  |
| Farming system |  |  |  | Farming system |  |  |  |
| Extensive | 1335 | 316 (23.6) | **3.3;1;0.066** | Extensive | 152 | 10 (6.5) | - |
| Intensive | 97 | 31 (31.9) |  | - | - |  |  |
| Production type |  |  |  | Production type |  |  |  |
| Beef production | 215 | 85 (39.5) | **29.5;1;0.000** | Beef production | 21 | 3 (14.2) | **2.3;1;0.125** |
| Dual purposes | 1217 | 262 (21.5) |  | Dual purposes | 131 | 7 (5.3) |  |
| Vector control |  |  |  | Babesiosis control |  |  |  |
| Yes | 358 | 73 (20.3) | **3.8;1;0.050** | Yes | 123 | 9 (7.3) | 0.5;1;0.450 |
| No | 1074 | 274 (25.5) |  | No | 29 | 1 (3.4) |  |
| Antiprotozoal drugs |  |  |  | Municipality |  |  |  |
| Yes | 307 | 55 (17.9) | **8.4;1;0.004** | Necoclí | 102 | 6 (5.8) | 2.3;1;0.621 |
| No | 1125 | 292 (25.9) |  | Turbo | 50 | 4 (8.0) |  |
| Municipality |  |  |  | - | - |  |  |
| Necoclí | 625 | 171 (27.3) | **50.1;4;0.000** | - | - |  |  |
| Turbo | 93 | 11 (11.8) |  | - | - |  |  |
| Saravena | 451 | 71(15.7) |  |  |  |  |  |
| Arauquita | 166 | 63 (37.9) |  |  |  |  |  |
| Tame | 97 | 31 (31.9) |  |  |  |  |  |
| *B. bovis* | | | | | | | |
| Age |  |  |  | Age |  |  |  |
| ≤ 1 year old | 358 | 96 (26.8) | **52.9;1;0.000** | ≤ 1 year old | 31 | 0 (0.0) | **8.4;1;0.004** |
| > 1 year old | 1074 | 111 (10.3) |  | > 1 year old | 121 | 27 (22.3) |  |
| Sex |  |  |  | Sex |  |  |  |
| Female | 1059 | 127 (11.9) | **18.6;1;0.000** | Female | 135 | 27 (20) | **4.1;1;0.042** |
| Male | 373 | 80 (21.4) |  | Male | 17 | 0 (0.0) |  |
| Breed |  |  |  | Breed |  |  |  |
| BI | 740 | 101 (13.6) | 1.0;2;0.575 | Carabao | 34 | 3 (8.8) | **6.9;3;0.075** |
| BI x BT | 297 | 48 (16.1) |  | Murra | 30 | 8 (26.6) |  |
| BT | 395 | 58 (14.6) |  | Mediterranean | 37 | 10 (27.0) |  |
|  |  |  |  | Crosses | 51 | 6 (11.7) |  |
| PCV |  |  |  | PCV |  |  |  |
| Anemic | 123 | 12 (9.7) | **2.4;1;0.177** | Anemic | 51 | 10 (19.6) | 0.1;1;0.672 |
| Normal | 1256 | 184 (14.6) |  | Normal | 101 | 17 (16.3) |  |
| Sampling season |  |  |  | Sampling season |  |  |  |
| Dry | 466 | 61 (13.0) | 1.3;2;0.512 | Dry | 57 | 11 (19.2) | 0.9;2;0.644 |
| Wet | 463 | 67 (14.4) |  | Wet | 51 | 7 (13.7) |  |
| Late wet | 503 | 79 (15.7) |  | Late wet | 44 | 9 (20.4) |  |
| Deparment |  |  |  | Deparment |  |  |  |
| Antioquia | 717 | 104 (14.5) | 0.0;1;0.839 | Antioquia | 152 | 27 (17.7) | - |
| Arauca | 715 | 102 (14.2) |  | - | - |  |  |
| Farming system |  |  |  | Farming system |  |  |  |
| Extensive | 1335 | 191 (14.3) | 0.3;1;0.554 | Extensive | 152 | 27 (17.7) | - |
| Intensive | 97 | 16 (16.4) |  | - | - |  |  |
| Production type |  |  |  | Production type |  |  |  |
| Beef production | 215 | 38 (17.6) | **2.0;1;0.145** | Beef production | 21 | 5 (23.8) | 0.61;1;0.435 |
| Dual purposes | 1217 | 169 (13.8) |  | Dual purposes | 131 | 22 (16.7) |  |
| Vector control |  |  |  | Babesiosis control |  |  |  |
| Yes | 358 | 55 (15.3) | 0.3;1;0.573 | Yes | 123 | 19 (15.4) | **2.3;1;0.124** |
| No | 1074 | 152 (14.1) |  | No | 29 | 8 (27.5) |  |
| Antiprotozoal drugs |  |  |  | Municipality |  |  |  |
| Yes | 307 | 32 (10.4) | **5.1;1;0.023** | Necoclí | 102 | 14 (13.7) | **3.3;1;0.063** |
| No | 1125 | 175 (15.5) |  | Turbo | 50 | 13 (26.0) |  |
| Municipality |  |  |  | - | - | - |  |
| Necoclí | 625 | 101 (16.1) | **13.1;4;0.030** | - | - | - |  |
| Turbo | 93 | 4 (4.3) |  | - | - | - |  |
| Saravena | 451 | 59 (13.0) |  |  |  |  |  |
| Arauquita | 166 | 27 (16.2) |  |  |  |  |  |
| Tame | 97 | 16 (16.4) |  |  |  |  |  |
| Note: Values in bold represent the variable that showed a *P < 0.25* according to the χ2 test and were included in the GEE analysis; (PCV) = packed cell volume; (VBP) = Vector borne pathogens; Given its identical frequency, variables (vector control) and (antiprotozoal drugs) were unified as babesiosis control in buffaloes. | | | | | | | |
